# Brachial artery thrombosis in a dog causing monoparesis mimicking nerve sheath tumor

**DOI:** 10.1111/jvim.16213

**Published:** 2021-07-13

**Authors:** Melissa N. Andruzzi, Andra K. Voges, Karen E. Russell, Nick D. Jeffery

**Affiliations:** ^1^ Department of Small Animal Clinical Studies Texas A&M University College Station Texas USA; ^2^ Department of Large Animal Clinical Studies Texas A&M University College Station Texas USA; ^3^ Department of Veterinary Pathobiology Texas A&M University College Station Texas USA

**Keywords:** arterial thromboembolism, clopidogrel, rivaroxaban, root signature

## Abstract

There are few differential diagnoses for non‐orthopedic thoracic limb lameness in adult dogs aside from nerve tumors and disk‐associated nerve compression; this report introduces another etiology. A 9‐year‐old male castrated mixed dog presented with an episodic history of nonweight‐bearing thoracic limb lameness. Additional clinical signs included an atrophied thoracic limb with cool paw pads and painful axillary region. Magnetic resonance imaging, computed tomography, ultrasound, and exploratory surgery confirmed a chronic thrombus of the right brachial artery. No underlying cause for the thrombus was identified. The dog has been successfully managed on long‐term rivaroxaban and clopidogrel. Follow‐up ultrasound of the thrombus suggested early remodeling.

**Abbreviations**
CT: computed tomography
EMG: electromyography
FXa: factor Xa

## 1 INTRODUCTION

Differential diagnoses for chronic thoracic limb lameness in adult dogs usually include both musculoskeletal and nonmusculoskeletal conditions.^1^, ^2^ Once degenerative joint disease, bicipital tendonitis, and musculoskeletal neoplasia have been ruled out, there are few common musculoskeletal diagnoses that remain and attention is then redirected to neurologic conditions. These conditions are most notably nerve sheath tumors and nerve compression secondary to lateralized intervertebral disk herniations, but also can include cervical spondylomyelopathy, cervical diskospondylitis, myopathies, and traumatic neuropathies.^2^, ^3^, ^4^, ^5^

In the early stages, neurologic causes of thoracic limb lameness might not be associated with appreciable neurologic deficits. Rather, nonspecific clinical signs, such as pain and muscle atrophy, often accompany the lameness and precede neurologic deficits.^1^, ^4^, ^5^, ^6^ In advanced stages of the disease, paresis, proprioceptive deficits, and reduced spinal reflexes become more evident and clearly imply a neurologic condition.^1^, ^6^, ^7^ However, for peripheral nerve tumors, the delay that occurs to allow these signs of neurologic dysfunction to manifest renders a poor prognosis, given the poor prognosis for treatment once spinal canal invasion has occurred.^4^

Early diagnostics that are often utilized in the workup of a nonmusculoskeletal thoracic limb lameness of adult dogs include electrodiagnostics and cross‐sectional imaging. Electrodiagnostics, specifically electromyography (EMG), nerve conduction studies, and F‐wave analysis, can be particularly helpful to differentiate disuse atrophy from neurogenic atrophy, and to localize which nerves might be involved in the brachial plexus.^2^, ^4^, ^8^

In this report, we describe a nonmusculoskeletal and, surprisingly, nonprimary neurologic cause of chronic thoracic limb lameness in an adult dog that shared many clinical characteristics of common differential diagnoses for thoracic limb lameness. This report describes a case of a nontraumatic in situ thoracic limb arterial thrombus in an adult dog and describes long‐term management of a chronic arterial thrombus in a thoracic limb using multimodal antithrombotic treatment.

## 2 CASE HISTORY

A 9‐year‐old male castrated mixed breed dog was presented to the Texas A&M Small Animal Teaching Hospital for a third episode of nonweight‐bearing lameness and apparent pain in the right thoracic limb. The first and second episodes were of similar character and had occurred 8 and 6 months previously; both previous episodes were managed elsewhere and had resolved with a week of medical management with oral analgesics and anti‐inflammatory medications. However, because this third episode had not responded to similar medical management strategies, the dog was referred to Texas A&M for further evaluation.

On presentation, the dog had normal vital measurements and a grade 4/6 left apical systolic heart murmur that had not been previously reported. The dog exhibited a nonweight‐bearing lameness of the right thoracic limb and held this limb in flexion at rest. Pain was elicited on light palpation of the right axillary region, right triceps muscle, and right thoracic limb paw pads. The distal extremities of this limb were thought to be cooler in comparison to the other 3 limbs.

Additional findings on orthopedic consultation included mild scapular muscle atrophy and moderate atrophy of the distal musculature, but there was no apparent orthopedic abnormality to account for the current clinical signs. Neurologic examination detected normal proprioception and limb reflexes and aside from root signature of the right thoracic limb and pain on palpation of the right caudal cervical area, no other abnormalities were found. Additional preliminary diagnostics included a CBC, chemistry panel (creatine kinase not included), and 3‐view thoracic radiographs, all of which were normal with the exception of increased serum globulin concentration (4.3 g/dL [reference interval, 1.7‐3.8 g/dL]).

Given concern for a lesion involving the right axillary region or a right‐sided radicular lesion of the C6‐T2 spinal cord segments, top differential diagnoses included neoplasia (peripheral nerve sheath tumor or other soft tissue sarcoma with secondary compression of brachial plexus nerves and vessels) and a lateralized intervertebral disk herniation.

**FIGURE 1:**
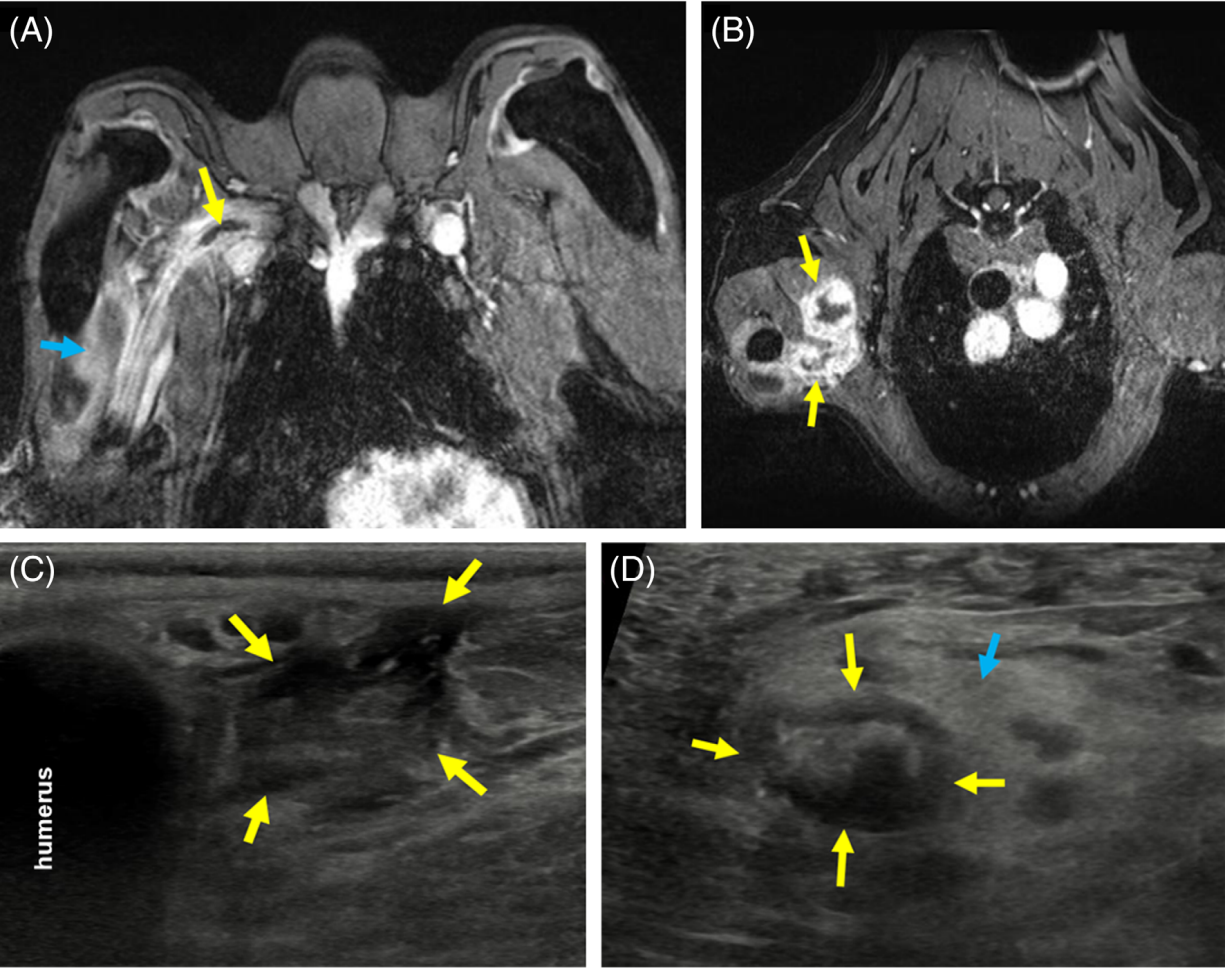
Images at first presentation. A, Postcontrast dorsal plane rapid gradient echo (RAGE) (T1‐weighted) MR image showing a hypointense structure within the right brachial arterial lumen (yellow arrow). There is contrast enhancement of the right triceps muscle (blue arrow). B, Postcontrast transverse plane RAGE (T1‐weighted) MR image showing heterogenous, centrally isointense, strongly peripherally contrast enhancing masses within the right triceps musculature (yellow arrows). C, Transverse ultrasound image of the right proximal brachium. There is disruption of the fiber pattern within the triceps musculature with overall hypoechogenicity (yellow arrows). D, Transverse ultrasound image of the right brachial artery, revealing an echogenic structure within the lumen of the proximal right brachial artery that contains hyperechoic foci with shadowing suggesting mineralization (yellow arrows). This artery lacked Doppler flow (not shown). The fascia surrounding this portion of the brachial artery was hyperechoic (blue arrow). MR, magnetic resonance

**FIGURE 2:**
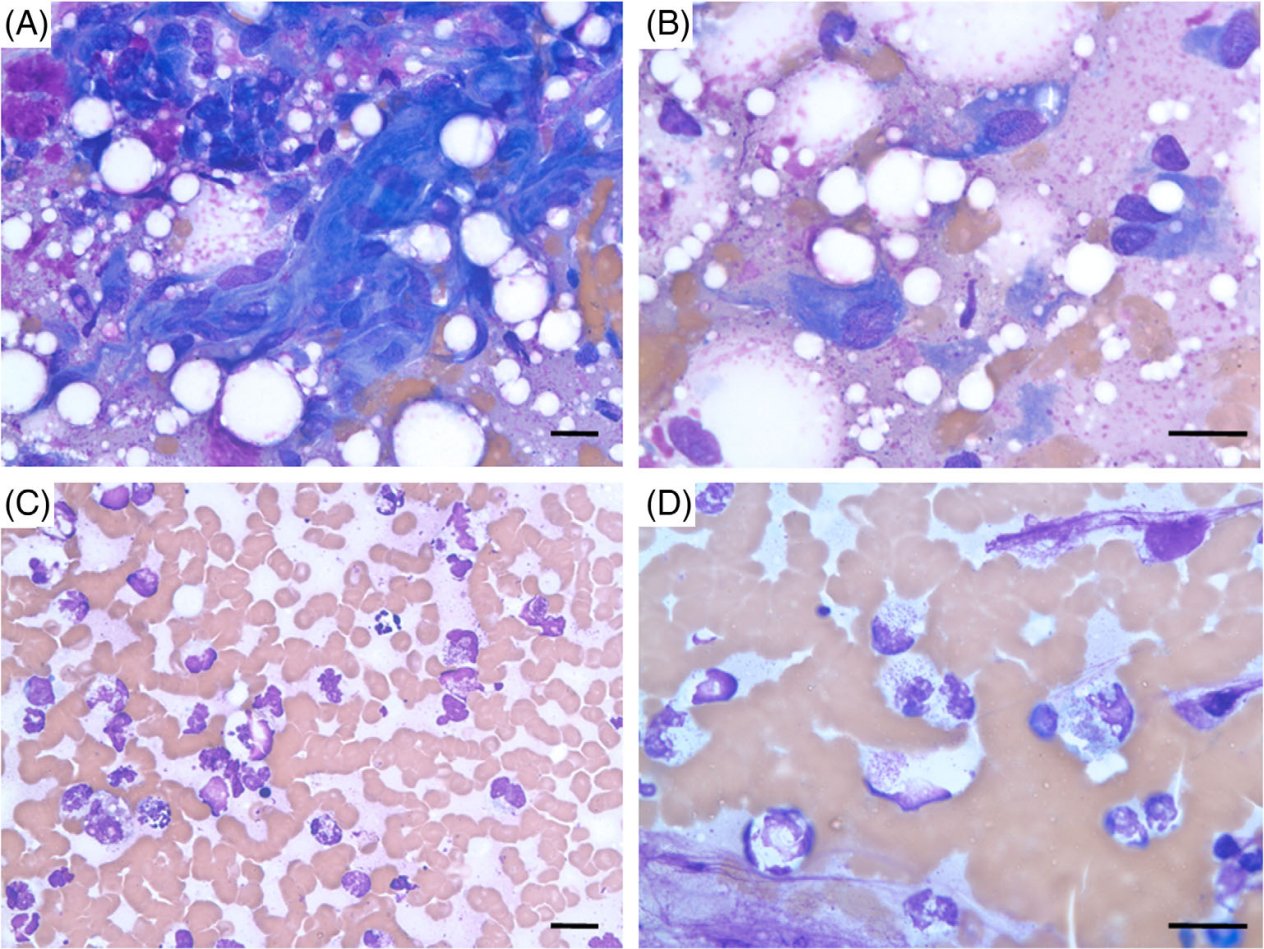
Images of intraoperative aspirates (Wright's stain). A,B, Cytology of aspirate of abnormal tissue adjacent to the brachial artery at the level of thrombus. A mesenchymal population is the predominant cell type, found as both aggregates and single cells. These cells display moderate anisocytosis and anisokaryosis and vary from spindled to occasionally oval or stellate in shape. The cytoplasm is dark blue and has a wispy appearance that trails away from the cell. The cells have an oval nucleus and nucleoli are often faint but appear round to slightly angular and 1 to 3 nucleoli are seen. C,D, Cytology of an aspirate of the brachial artery thrombus. There are many nucleated cells amid a background of erythrocytes and a light pink, granular background with cellular debris and nuclear streaming. High numbers of degenerate neutrophils and a few macrophages are noted. The neutrophils often contain small, punctate, basophilic structures that are presumed to be bacterial short rods or cocci. Scale bars: 15 μm in all images

Under general anesthesia, EMG revealed patchy areas of positive sharp waves and fibrillation potentials in the biceps, triceps, and extensor carpi radialis muscles, indicative of a myopathy and/or denervation. Nerve conduction studies and F‐wave analysis were not performed because extensive imaging of this area was planned to follow. Magnetic resonance imaging, computed tomography (CT), and ultrasound of the right brachial plexus were performed under the same anesthetic event (Figure 1). An extensive thrombus of the right brachial artery was found, with hyperechoic and shadowing material that was compatible with mineralization of the thrombus, suggesting chronicity. Additionally, there were mass‐like lesions within the right triceps and biceps brachii muscles and multifocal intramuscular lesions in the surrounding right axillary musculature; priority was given to an ischemic process although neoplastic infiltration could not be ruled out. Ultrasound‐guided cytology of the muscular lesions was inconclusive (Figure 2). Computed tomography angiogram of the abdomen found no evidence of other thromboses, neoplasia, and/or other intramuscular lesions. An echocardiogram was recommended to rule out cardiac disease as a cause for thromboembolic disease, but was declined by the owner. Viscoelastic coagulation monitoring revealed appropriate coagulation status. Clopidogrel (1.25 mg/kg PO q24h) was initiated.

Exploratory surgery of the right axillary region was performed the next day. Intraoperatively, the mass‐like lesions within the biceps brachii and triceps muscles were not grossly visible or distinctly palpable, so sampling was not attempted. However, the right brachial artery had a palpably large and firm intraluminal mass, with strong pulses present proximally but absent distally. Abnormally pale, firm, haphazardly deposited, soft tissue was noted adjacent to the brachial artery, at the level of the presumed thrombus. The thrombus was aspirated for cytology (Figure 2), which raised concern for septic neutrophilic inflammation but repeat cytologic evaluation raised reasonable doubt for intracellular bacteria; aspirates were also submitted for aerobic and anaerobic cultures, which ultimately returned no growth.

The adjacent abnormal soft tissue was sampled for cytology and histopathology. While the cytologic findings were compatible with sarcoma (primary considerations given to a peripheral nerve sheath tumor, fibrosarcoma, or liposarcoma), subsequent histopathology showed no evidence of neoplastic cells and instead confirmed a chronic, diffuse, neutrophilic, and lymphoplasmacytic perivascular inflammation with focal vasculitis and thrombosis.

Rivaroxaban (1.3 mg/kg PO q24h) was added into the therapeutic regimen and the clopidogrel dosage was increased to 2.5 mg/kg PO q24h. The dog's pain began resolving in hospital and it was discharged shortly thereafter. Two weeks after discharge, the dog was reportedly bearing weight on the right thoracic limb and 6 weeks later, the dog was reportedly normal at home. At that time, the rivaroxaban dose was halved (to 0.6 mg/kg/d) due to cost concerns and the clopidogrel dose remained constant. Six‐month follow‐up confirmed the dog to still be doing well at home with no clinical signs.

**FIGURE 3:**
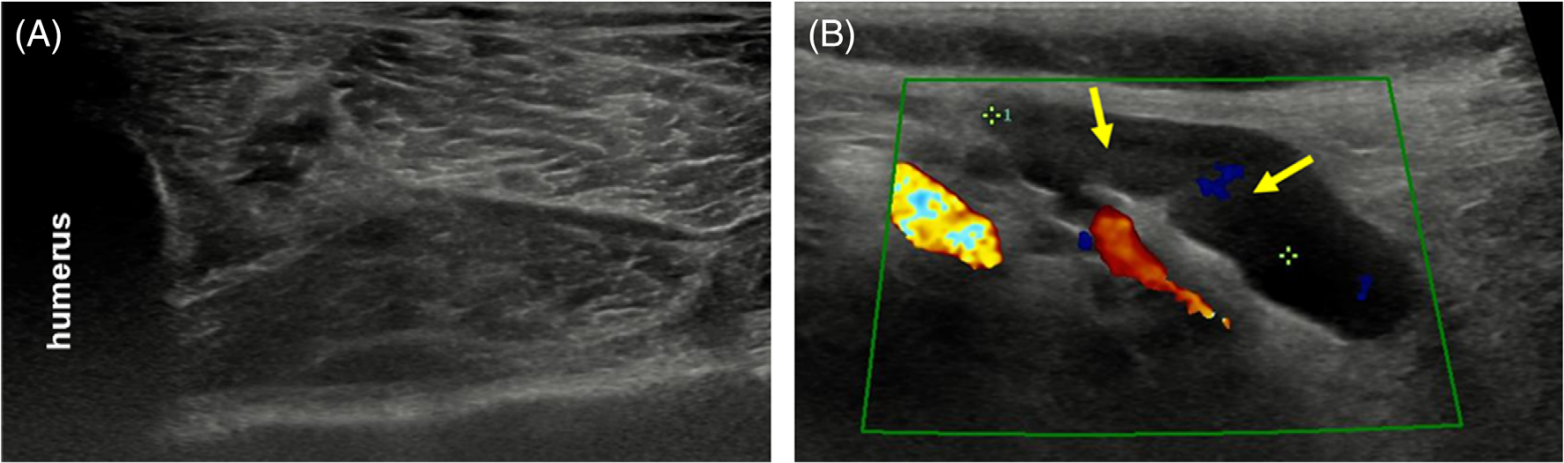
Images at 8‐month re‐examination. A, Transverse ultrasound image of the right proximal brachium. There is now normal fiber pattern and echogenicity in the triceps muscle. B, Sagittal Doppler ultrasound image of the right brachial artery in the axilla. There remains reduced to absent Doppler flow in the brachial artery at this site but flow was identified more distally in the limb (not shown). There is an ill‐defined echogenic structure within the brachial artery lumen (yellow arrows) and the previously noted hyperechoic foci of mineralization are no longer apparent

Eight months after initial diagnosis, the dog returned for a repeat ultrasound to evaluate the thrombus. Neurologic and physical examinations were both unremarkable. Ultrasound confirmed a persistent thrombus within the proximal brachial artery, although the previously identified intralesional mineralization was not apparent (Figure 3). Additionally, the biceps and triceps musculature lesions suspected to be areas of ischemia had resolved. Prothrombin time (PT) and partial thromboplastin time (PTT) were tested and were within normal limits. No changes to the therapeutic plan were made and the dog remains on rivaroxaban and clopidogrel indefinitely.

## 3 DISCUSSION

This case report highlights a previously unreported etiology for a rather commonplace clinical presentation that historically has limited differential diagnoses.^1^, ^2^, ^3^ The most common differential diagnoses for a nonorthopedic, chronic, thoracic limb lameness in an adult dog include neoplasia of the nerve roots and a lateralized disk herniation with nerve root compression.^1^, ^2^, ^3^ Diagnosis can often be a prolonged process because many of these disease processes can have insidious clinical signs^1^, ^4^, ^5^, ^6^ and, anecdotally, often respond transiently to empirical anti‐inflammatory and analgesic treatment.

In this particular case, the definitive diagnosis of a chronic thrombus was associated with a good prognosis and by example, this case might motivate clinicians to rule out this disease process as an etiology for this type of clinical presentation. However, there are several features of the canine arterial thrombi, including chronicity of the disease process, lack of underlying inciting factors in many cases, and overall perceived rarity of this disease process in dogs, that might make the diagnosis challenging.

Clinical signs of arterial thrombosis in dogs can be chronic. In several retrospective studies^9^, ^10^, ^11^ on arterial thrombosis (most of which were aortic in origin), clinical signs ranged from 2 hours to 12 months, with median duration of clinical signs ranging from 1 to 2 months. In 1 report, only 22% had a peracute onset of paralysis of limb(s) without previous signs of dysfunction or lameness.^10^ Generally, it appears that aortic thromboembolism in dogs has 2 separate phenotypes—1 characterized by acute onset that manifests with severe limb dysfunction and is associated with a shorter survival time and the second characterized by chronicity with waxing and waning lameness, exercise intolerance, and behavior suggesting episodic pain. It is assumed that collateral circulation explains the toleration of a chronic, partially obstructive arterial thrombus.^9^

Another factor in complicating diagnosis of arterial thrombi in dogs is that underlying disease processes are oftentimes not found; many canine arterial thrombi occur in situ as a primary thrombus, rather than being the consequence of a thromboembolic event, with no obvious inciting factor. In a retrospective study of 26 dogs with arterial thrombi, 58% had no concurrent disease conditions at time of diagnosis.^11^ Of dogs that do have an underlying pro‐thrombotic disease, protein‐losing diseases (mostly protein‐losing nephropathies), neoplasia, and hyperadrenocorticism are most commonly reported. Coagulation panels and clotting times of these dogs are often normal although increased fibrin‐degradation products and D‐dimers might occasionally be detected.^9^, ^11^, ^12^, ^13^ In dogs found to have structural heart disease at the time of arterial thrombus diagnosis, endocardiosis is reportedly most common, which is unsurprising given its prevalence and also casts doubt on its clinical importance.^9^, ^12^

It is possible that brachial artery thrombosis is underdiagnosed in dogs and it is certainly uncommonly reported. The only reported cases include a brachial artery thrombus secondary to a traumatic humeral fracture^14^ and presumptive thoracic limb arterial thromboembolisms secondary to endocarditis.^15^ The infrequency of reports of thoracic limb arterial thrombi in dogs might be because this disease process can easily masquerade as other etiologies (such as degenerative joint disease, lateralized disk extrusion, or nerve sheath tumor) or because less accessible diagnostics, such as electrodiagnostics and advanced imaging modalities, are required to confirm such a diagnosis. In this case, electrodiagnostics suggested a neurologic condition, rather than the primary circulatory problem that was ultimately diagnosed. It was hypothesized that the EMG abnormalities were caused by poor perfusion.^5^, ^8^ However, this suggests that advanced imaging must be paired with electrodiagnostics to properly investigate this disease process. Additionally, given that arterial thrombi tend to occur in adult dogs, it might be less motivating for owners to pursue advanced diagnostics over empirically treating for more common conditions such as osteoarthritis or neoplasia.

However, although advanced diagnostics were required to reach a diagnosis in this case, the treatment was noninvasive, relatively affordable, and administered on an outpatient basis. Medical treatment of thrombi in dogs is directed at preventing new thrombus formation and breaking down the current thrombus. There are also newer minimally invasive approaches reported for arterial thrombosis, such as vascular stenting, percutaneous mechanical thrombectomy, intra‐arterial catheter‐directed thrombolysis, and ultrasound‐assisted thrombolysis, but these case reports are sporadic, of low power, and have variable results.^16^, ^17^, ^18^, ^19^, ^20^

In this particular case, clopidogrel and rivaroxaban were used together with the aim of prophylaxis against future thrombogenesis, as well as thrombolysis of the current thrombus in hopes of re‐establishing a patent arterial lumen.^21^ Although a recent consensus statement described the evidence for use of 8 antithrombotic drugs,^21^ there is no current agreement on whether treating acute or chronic thrombosis should be governed by different principles and as such, this case was managed similarly to an acute thrombotic event. We acknowledge that our multimodal antithrombotic approach is largely presumptive and anecdotal, as there is no current evidence to suggest that single antithrombotic treatment is less efficacious.

Clopidogrel, a platelet aggregation inhibitor, was chosen, given that recent reports dictate that prophylactic treatment of arterial thrombogenesis should include antiplatelet drugs, because arterial thrombi tend to contain a greater number of platelets than venous thrombi.^18^, ^21^ However, there is no current evidence that clopidogrel is superior to aspirin for the treatment of arterial thromboembolism in dogs, and aspirin could have been chosen instead. Rivaroxaban is a factor Xa (FXa) inhibitor; FXa is a propagator of thrombin formation well as a potent platelet agonist (by means of activating protease‐activated receptor‐1 [PAR‐1]) and thus is a key player in driving arterial thrombosis.^22^ There is currently 1 case series describing the efficacy of rivaroxaban, which consists of 4 dogs with arterial (n = 3) and venous (n = 1) thromboses. Improvement (defined as reduction in thrombus size and reduction or resolution of clinical signs) was temporally associated with rivaroxaban administration in dogs after switching from other antithrombotics.^20^ Rivaroxaban administration produces a less dense and compact thrombus structure, with reduced platelet and fibrinogen deposition.^22^ On the 8‐month follow‐up ultrasound of our case, the thrombus was no longer noted to show the hyperechoic and had lost the previously observed shadowing properties, perhaps suggesting remodeling or early dissolution of the thrombus.

Future management of this case entails long‐term continuation of both clopidogrel and rivaroxaban. This is based on the recent consensus statement on discontinuation of antithrombotic treatment, which recommends indefinite antithrombotic treatment in patients in which the underlying precipitating condition for in situ arterial thrombus formation is unknown.^23^

This case report has several limitations. In the initial evaluation, a creatine kinase value and differential lactate and blood pressures of the affected and nonaffected limbs were not performed given the low suspicion for thoracic limb thrombus at that time, but might have been useful to suggest a vascular lesion earlier in the case investigation. Second, after diagnosis of an arterial thrombus, etiologies of thromboembolic disease, such as a proteinuria and endocrine disease, could have been more vigorously investigated with diagnostics such as a urine protein : creatinine ratio and thyroid and adrenal testing. However, given the lack of systemic clinical signs and lack of supporting abnormalities on screening bloodwork that would suggest a protein‐losing disease or endocrinopathy, these diagnostics were not pursued in this specific case but may be helpful in the investigation of future similar cases. This case is also limited by a lack of histopathology of the brachial artery thrombus that definitively confirmed a purely in situ thrombus, rather than (albeit less likely) a neoplasia of the arterial wall. Additionally, most reports on arterial thrombi in dogs are of aortic thromboembolism, either to the distal aorta or pulmonary artery, so direct comparisons to this particular case are difficult. Finally, as stated previously, there is a paucity of literature to guide thrombotic treatment in dogs and newer agents such as rivaroxaban are inadequately studied.

## CONFLICT OF INTEREST DECLARATION

Authors declare no conflict of interest.

## OFF‐LABEL ANTIMICROBIAL DECLARATION

Authors declare no off‐label use of antimicrobials.

## INSTITUTIONAL ANIMAL CARE AND USE COMMITTEE (IACUC) OR OTHER APPROVAL DECLARATION

Authors declare no IACUC or other approval was needed.

## HUMAN ETHICS APPROVAL DECLARATION

Authors declare human ethics approval was not needed for this study.
